# Gut and skin dysbiosis in acne vulgaris: a comparative microbiome analysis

**DOI:** 10.3389/fcimb.2026.1904271

**Published:** 2026-08-06

**Authors:** Agata Lesiak, Agnieszka Krawczyk, Justyna Piasta, Jakub Spałek, Joanna Szajkowska, Ewa Papros, Beata Kręcisz, Bonita Durnaś, Tomasz Gosiewski, Robert Bucki

**Affiliations:** 1 Institute of Medical Science, Collegium Medicum, Jan Kochanowski University, Kielce, Poland; 2 Department of Microbiology, Provincial Combined Hospital in Kielce, Kielce, Poland; 3 Department of Molecular Medical Microbiology, Chair of Microbiology, Jagiellonian University Medical College, Kraków, Poland; 4 Dermatology Clinic, Provincial Combined Hospital in Kielce, Kielce, Poland; 5 Department of Clinical Microbiology, Holy-Cross Oncology Center of Kielce, Kielce, Poland; 6 Microbiome Research Laboratory, Department of Molecular Medical Microbiology, Chair of Microbiology, Faculty of Medicine, Jagiellonian University Medical College, Kraków, Poland; 7 Department of Medical Microbiology and Nanobiomedical Engineering, Medical University of Białystok, Białystok, Poland

**Keywords:** acne vulgaris, skin microbiota, gut microbiota, next-generation sequencing (NGS), gut -skin axis, dysbiosis

## Abstract

**Introduction:**

Acne vulgaris (AV) is a chronic inflammatory skin disorder with multifactorial etiology involving host–microbiome interactions. While cutaneous dysbiosis has been extensively studied, the contribution of the gut microbiome and its potential interaction with the skin microbiome remains insufficiently characterized.

**Methods:**

In this pilot study, paired facial skin swabs and stool samples were collected from 17 patients with moderate-to-severe AV and 14 healthy controls. Microbial DNA was extracted and the V3–V4 regions of the bacterial 16S rRNA gene were sequenced on the Illumina MiSeq platform.

**Results:**

Acne patients exhibited significantly reduced gut microbial alpha diversity compared to controls (p < 0.001 across indices), accompanied by decreased diversity in the skin microbiome. Beta diversity analyses revealed distinct gut microbial community structures between groups, whereas skin microbial composition showed more subtle differences. Taxonomic profiling identified pronounced alterations in gut microbiota, contrasting with relatively modest shifts in skin communities.

**Conclusions:**

Our findings reveal a systemic pattern of microbial dysbiosis in AV, characterized by marked alterations in the gut microbiome alongside concurrent, albeit less pronounced, changes in the skin microbiome. These results support a role for the gut–skin axis in acne pathophysiology and highlight the need for integrative, system-level analyses of host–microbiome interactions in inflammatory skin diseases.

## 1 Introduction

Acne vulgaris (AV) is one of the most common skin diseases affecting adolescents and young adults. It affects approximately 85% of teenagers; however, it may occur across all age groups and persist into adulthood. According to the 2010 Global Burden of Disease study, AV was the fourth most prevalent skin disorder worldwide. It was estimated that AV affected approximately 8.96% of men and 9.81% of women globally (Heng and Chew, 2020). Although AV is not associated with mortality, it is frequently linked to significant psychological and physical burdens, including scarring, low self-esteem, depression, and anxiety disorders (Hay et al., 2014; Zaenglein et al., 2016). Clinically, AV is considered as a chronic and multifactorial inflammatory disease of the pilosebaceous unit (PSU), a structure composed of the hair follicle, sebaceous gland, and arrector pili muscle, and is associated with excessive sebum production (hyperseborrhea) (Lesiak et al., 2024). From a microbiological perspective, AV is characterized by imbalanced skin microbial colonization (dysbiosis), particularly involving *Cutibacterium acnes* (*C. acnes*) (Ramasamy et al., 2019). Following the taxonomic reclassification proposed by Scholz and Kilian (2016), the species formerly known as *Propionibacterium acnes* is currently classified as *Cutibacterium acnes* (Scholz and Kilian, 2016). Although the former nomenclature is still encountered in the literature (Alexeyev et al., 2018), the currently accepted taxonomic designation, *Cutibacterium acnes*, is used throughout this manuscript. Current knowledge about the pathogenesis of acne is constantly evolving. Key pathogenic factors include follicular hyperkeratinization, microbial colonization with *C. acnes*, sebum production, and complex inflammatory mechanisms involving both innate and adaptive immunity. In addition, neuroendocrine regulatory mechanisms, diet, and genetic and nongenetic factors may contribute to the multifactorial process of AV pathogenesis (Clatici et al., 2018). The skin microbiota coexists with the human host in a symbiotic or commensal relationship, maintaining an eubiosis state that supports the skin’s nonspecific defense barrier. In particular, *C. acnes* and *Staphylococcus epidermidis* play critical roles in skin homeostasis. Dysbiosis, manifested by qualitative and/or quantitative alterations in microbial composition, is a hallmark of several skin disorders, including AV (Condrò et al., 2022; Dreno et al., 2024). However, growing evidence suggests that AV should be considered not only as a localized skin disorder but also as a condition influenced by systemic factors, including the intestinal microbiome (Tang et al., 2025). In recent years, the concept of the gut–skin axis has gained increasing attention. The intestinal microbiota plays a fundamental role in immune modulation, metabolic regulation, and maintenance of epithelial barrier integrity (Salem et al., 2018). Dysbiosis of the gut microbiome has been associated with various dermatological conditions, including AV, atopic dermatitis, psoriasis, and rosacea. Alterations in gut microbial composition may contribute to systemic low-grade inflammation, increased intestinal permeability, and immune dysregulation, which in turn can influence skin homeostasis and inflammatory responses (De Pessemier et al., 2021). Parallel to intestinal dysbiosis, disturbances in the skin microbiome are increasingly recognized as an important factor in acne development. Modern sequencing techniques have demonstrated that acne is not solely associated with overgrowth of specific bacterial species, but rather with changes in microbial diversity and community structure (Dreno et al., 2024). Nevertheless, most existing studies focus exclusively on either the gut or the skin microbiome, while investigations addressing both ecosystems simultaneously in the same individuals remain limited. High-throughput sequencing of the bacterial 16S rRNA gene has become a standard approach for comprehensive characterization of microbial communities in clinical studies (Johnson et al., 2019). This technique enables culture-independent identification of microbial taxa and allows comparison of microbiome composition across different body sites and clinical conditions. In particular, paired analysis of skin and stool samples provides a unique opportunity to explore interactions between intestinal and cutaneous microbial ecosystems within individual subjects. Therefore, the aim of this pilot study was to characterize and compare the gut and skin microbiome profiles in patients with AV and healthy controls using 16S rRNA gene sequencing. By analyzing paired stool and facial skin samples, we sought to explore potential alterations in microbial diversity and taxonomic composition along the gut–skin axis in acne. The results of this study may contribute to a better understanding of the role of microbiome dysbiosis in acne pathogenesis and provide a foundation for future larger-scale investigations.

## 2 Materials and methods

### 2.1 Characteristics of the study design and participants

This pilot study included a total of 31 participants aged 18–35 years, including both women and men. The study group consisted of 17 patients with acne AV by a dermatology specialist. The control group comprised 14 healthy volunteers without a history of acne or other chronic dermatological conditions. Inclusion criteria for the study group were a clinical diagnosis of moderate-to-severe AV and age over 18 years. Exclusion criteria for all participants included gastrointestinal diseases, long-term antibiotic therapy, probiotic supplementation within three months prior to sample collection, and chronic systemic disorders. Written informed consent was obtained from all participants prior to enrollment. Ethical approval for the study was obtained from the Bioethics Committee of the Collegium Medicum, Jan Kochanowski University in Kielce (approval No. 37/2022 and 7/2024). All study procedures were conducted in accordance with the principles of the Declaration of Helsinki.

### 2.2 Sample collection

For each participant, paired skin swab and stool samples were collected to enable individual-level comparison of the skin and gut microbiome. Skin swabs were collected from the entire facial skin using the eNAT collection system (COPAN, Italy), containing nucleic acid stabilization medium. Participants were instructed not to wash their face for approximately 12 hours prior to sampling. Additionally, on the day preceding sample collection, all participants washed their face in the morning and evening using only water and were instructed not to apply any cosmetics, creams, or other skincare products. Sampling was performed by gently rubbing the sterile swab over the facial skin for approximately 2 minutes. After collection, swabs were placed in the eNAT transport medium and stored at −80 °C until DNA extraction. Stool samples were self-collected by participants at home using stool collection tubes containing DNA stabilizer (Invitek Molecular, Germany), according to the manufacturer’s instructions. After collection, samples were delivered to the laboratory Department of Microbiology and Immunology, Collegium Medicum, Jan Kochanowski University in Kielce and stored at −80 °C until further processing.

### 2.3 DNA extraction, DNA quantification and quality assessment

Genetic material from skin swab samples was isolated using the QIAamp UCP Pathogen Mini Kit together with Pathogen Lysis Tubes S (Qiagen, Germany), according to the manufacturer’s instructions. Genetic material from stool samples was extracted using the PSP Spin Stool DNA Basic Kit (Invitek Molecular, Germany), following the manufacturer’s protocol. Extracted DNA was stored at −20 °C until further analysis. Following DNA extraction, nucleic acid concentration was measured using a Quantus™ Fluorometer (Promega, USA) with the QuantiFluor^®^ ONE dsDNA System (Promega, USA; excitation at 485 nm and emission at 530 nm), according to the manufacturer’s instructions. DNA purity was evaluated spectrophotometrically using a NanoDrop™ instrument (Thermo Scientific, USA) by measuring the A260/A280 ratio. A DNA purity threshold of ≥1.7 was considered acceptable, and all samples met this criterion.

### 2.4 Preparation of genomic library

Skin swab-derived DNA extracts yielded low biomass, with concentrations below 5 µg/mL. Therefore, to increase amplification sensitivity and specificity, a nested PCR (polymerase chain reaction) strategy was applied for skin-derived samples prior to library preparation. Amplification of the bacterial 16S rRNA gene encompassing the V3–V4 hypervariable regions was performed using a two-step nested PCR approach adapted from a previously validated low-biomass protocol (Sroka-Oleksiak et al., 2020). Region-specific primers compatible with Illumina sequencing were used. Negative controls (no-template reactions) were included at each PCR step to monitor potential contamination. Primers targeting the 16S rRNA gene V3 and V4 regions and the amplification programme are presented **Table 1**.

**Table 1:** Nested PCR protocol for amplification of the 16S rRNA V3–V4 regions.

PCR step	Primer designation	Primer sequence (5’–3’)	Reaction volume	Cycling conditions
First-round PCR	Forward (Inner-F)	ACGGCCNNRACTCCTAC ^1^	20 µL total	Initial denaturation: 95 °C – 5 min 30 cycles of: 95 °C – 15 s; 48 °C – 20 s; 72 °C – 30 s Final extension: 72 °C – 5 min
	Reverse (Inner-R)	TTACGGNNTGGACTACHV		
Second-round PCR	Forward (Outer-F)	CCTACGGGNGGCWGCAG ^2^	25 µL total	Initial denaturation: 95 °C – 5 min 25 cycles of: 95 °C – 30 s; 55 °C – 30 s; 72 °C – 30 s Final extension: 72 °C – 5 min
	Reverse (Outer-R)	GACTACHVGGGTATCTAATCC		

The overhang adapter sequences (F: TCGTCGGCAGCGTCAGATGTGTATAAGAGACAG and R: GTCTCGTGGGCTCGGAGATGTGTATAAGAGACAG) were added to the internal primer attached to the 5′ end. 1 Externall primers. 2 Internal primers.

Following nested PCR amplification, DNA products were purified using the NucleoSpin^®^ Gel and PCR Clean-up Kit (Macherey-Nagel, Germany) according to the manufacturer’s protocol. The products of amplification were subjected to agarose gel electrophoresis to verify the size of the obtained amplicons. DNA extracted from stool samples was amplified using primers targeting the 16S rRNA V3 and V4 regions. In contrast to skin-derived samples, stool DNA was subjected to a single-step PCR amplification without a nested PCR approach, as sufficient DNA concentration was obtained following extraction. PCR products were verified by agarose gel electrophoresis to confirm the expected amplicon size. Amplicon libraries were prepared following the Illumina 16S Metagenomic Sequencing Library Preparation protocol, including purification, sample indexing, sample quantification, and pooling (Inc., 2013).

### 2.5 Next generation sequencing

Library concentration was measured using PicoGreen fluorescent dye (Thermo Fisher, Waltham, MA, USA) and pooled with 30% spike-in PhiX control DNA (Illumina, San Diego, USA) (Krawczyk et al., 2023). The pooled libraries were loaded onto a MiSeq Reagent Kit V3 cartridge (600 cycles) and sequencing was performed on an Illumina MiSeq platform at the Department of Microbiology and Immunology, Collegium Medicum Jan Kochanowski University in Kielce. Negative control (molecular-grade water instead of DNA) was included throughout the library preparation and next generation sequencing workflow. Raw sequencing data were demultiplexed and exported for downstream bioinformatic analysis.

### 2.6 Bioinformatics and statistical analysis

Raw paired-end sequencing reads were processed by trimming primer and adapter sequences from forward and reverse reads using Cutadapt. Quality filtering was performed by trimming bases with Phred quality scores below Q30, and reads shorter than 30 nucleotides after trimming were discarded. Taxonomic classification was conducted using the SILVA 138.2 SSU reference database, customized for the 16S rRNA V3–V4 region. Sequences corresponding to the V3–V4 region were extracted from the SILVA database based on the 341F/785R primer pair, and a Naive Bayes classifier was trained for taxonomic assignment. Sequence data were processed using an amplicon sequence variant (ASV) denoising approach, which reduces sequencing error and identifies unique sequence variants. The output consisted of an ASV abundance table for all samples and a set of representative ASV sequences. Taxonomic profiles were analyzed at successive taxonomic levels from kingdom to species. Relative abundances were visualized using bar plots. For clarity of visualization, taxa with relative abundance below 3% were grouped into a collective category labeled “Other <3%”. Differential abundance analysis between groups was performed at each taxonomic level using ALDEx2 with centered log-ratio (CLR) transformation. Statistical significance was assessed using the Kruskal–Wallis test. Alpha diversity was calculated using the following indices: Observed features, Shannon, Simpson, inverse Simpson, and Chao1. Normality of data distribution was assessed using the Shapiro–Wilk test. Depending on data distribution, either Student’s t-test or Wilcoxon rank-sum test was applied. Pairwise comparisons between study groups were performed. Beta diversity was assessed based on the Bray–Curtis distance metric. Distance matrices were visualized using hierarchical clustering heatmaps (average linkage) and sample similarity dendrograms. Differences in community composition between groups were evaluated using PERMANOVA (adonis) on Bray–Curtis distance matrices, with pairwise group comparisons. Additionally, principal component analysis (PCA) was performed on relative taxonomic abundance tables, and principal coordinates analysis (PCoA) was conducted on Bray–Curtis distance matrices to visualize clustering patterns among samples.

## 3 Results

### 3.1 Baseline characteristics of the study population

A total of 31 participants were analyzed, including 17 patients with moderate-to-severe AV and 14 healthy controls. The acne group comprised 13 females and 4 males, while the control group consisted of 9 females and 5 males. The mean age of the cohort was 26 years (range: 18–35 years).

### 3.2 DNA concentration and purity assessment

DNA concentrations obtained from stool samples ranged from 55 to 208 ng/µL, while skin-derived DNA concentrations were lower, ranging from 1 to 2, 5 ng/µL. Spectrophotometric assessment demonstrated A260/A280 ratios ≥1.7 in all samples, confirming adequate purity for subsequent PCR amplification and sequencing procedures.

### 3.3 Gut microbiome alpha diversity

**Figure 1:**
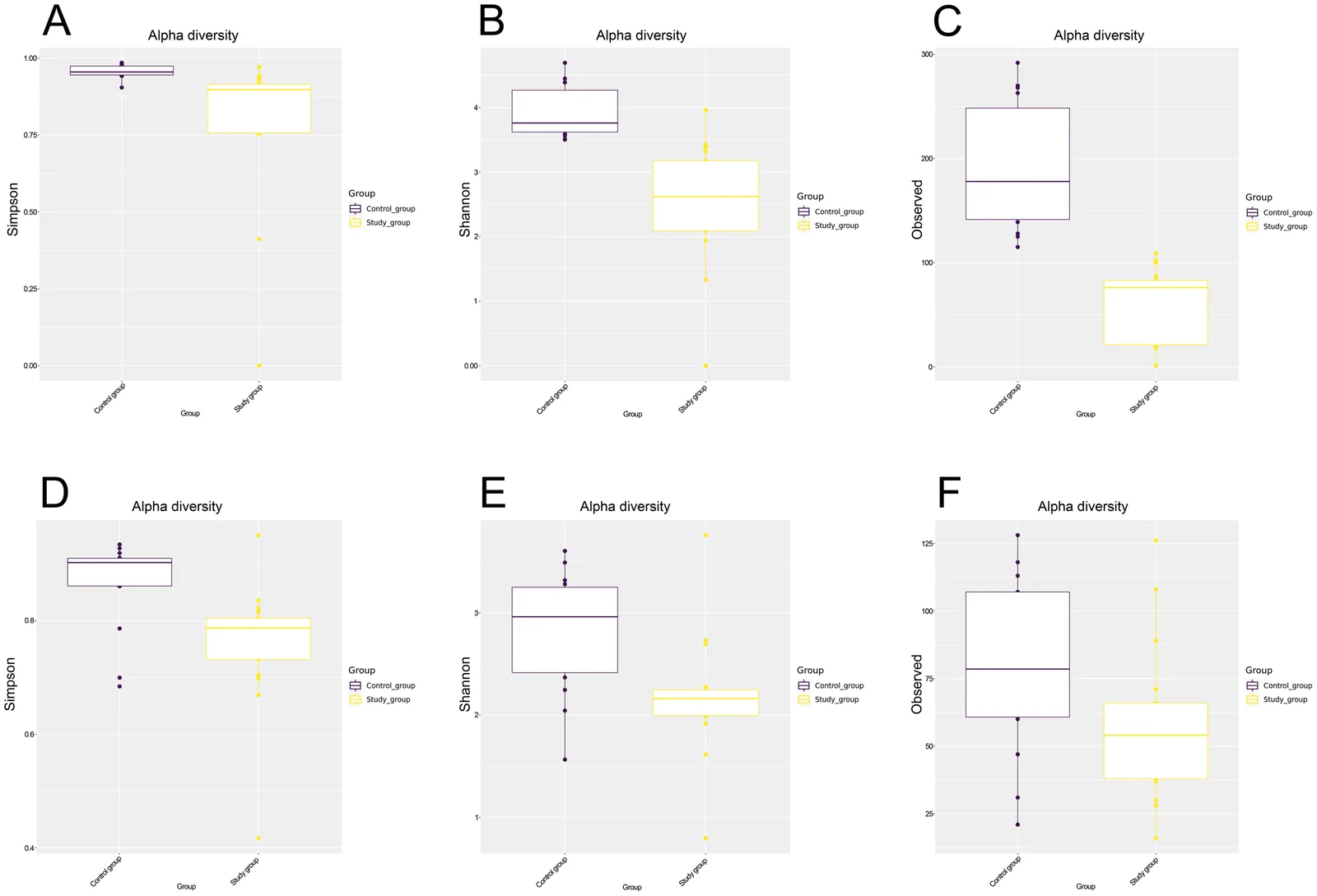
Alpha diversity of the gut and skin microbiome in control subjects and acne vulgaris patients. **(A–C)** show gut microbiome diversity measured using **(A)** Simpson index, **(B)** Shannon index, and **(C)** observed richness. **(D–F)** show the corresponding alpha diversity metrics for the skin microbiome.

Analysis of gut microbial alpha diversity demonstrated a statistically significant reduction in microbial richness and diversity in patients with AV compared with healthy controls (**Figures 1A–C**). The number of observed taxa was markedly lower in the acne group than in the control group (mean 62.71 vs. 190.87; *p* < 0.001). Similarly, the Chao1 index revealed significantly reduced estimated richness in patients with acne compared with controls (62.71 vs. 191.05; *p* < 0.001). Diversity indices accounting for both richness and evenness were also significantly decreased in the acne group. The Shannon diversity index was significantly lower in patients with acne than in healthy controls (2.46 vs. 3.93; *p* < 0.001). Likewise, the Simpson diversity index showed a significant reduction in the acne group (0.747 vs. 0.957; *p* < 0.001). Consistently, the inverse Simpson index was significantly lower in patients with acne compared with controls (9.64 vs. 29.56; *p* < 0.001), indicating reduced overall gut microbial diversity. All six alpha diversity metrics showed consistent patterns between groups in fecal samples; therefore, representative indices are shown in **Figure 1** for clarity.

### 3.4 Skin microbiome alpha diversity

Analysis of skin microbial alpha diversity revealed differences between patients with AV and healthy controls across multiple diversity indices (**Figures 1D–F**). All six alpha diversity metrics showed consistent patterns between groups in skin samples; therefore, representative indices are shown in **Figure 1** for clarity. The number of observed taxa tended to be lower in the acne group compared with controls (57.94 vs. 79.93), although this difference did not reach statistical significance (*p* = 0.06). Similarly, the Chao1 index indicated a trend toward reduced estimated richness in acne patients compared with controls (57.99 vs. 79.93; *p* = 0.06). In contrast, diversity indices incorporating both richness and evenness demonstrated statistically significant differences between groups. The Shannon diversity index was significantly lower in the acne group than in healthy controls (2.17 vs. 2.80; *p* = 0.006). Likewise, the Simpson diversity index was significantly reduced in patients with acne compared with controls (0.76 vs. 0.86; *p* = 0.004). Consistently, the inverse Simpson index was also significantly lower in the acne group (5.25 vs. 9.14; *p* = 0.004), indicating reduced overall skin microbial diversity.

### 3.5 Gut microbiome beta diversity

Gut microbial beta diversity was assessed using the Bray–Curtis distance metric across multiple taxonomic levels. Statistically significant differences in community composition between patients with AV and healthy controls were observed at the phylum level (*p* = 0.047) and at the species level (*p* = 0.025). At intermediate taxonomic ranks, differences in gut microbial community structure did not reach statistical significance, although a trend toward separation between groups was observed at the class (*p* = 0.051), order (*p* = 0.095), family (*p* = 0.11), and genus (*p* = 0.086) levels.

### 3.6 Skin microbiome beta diversity

**Figure 2:**
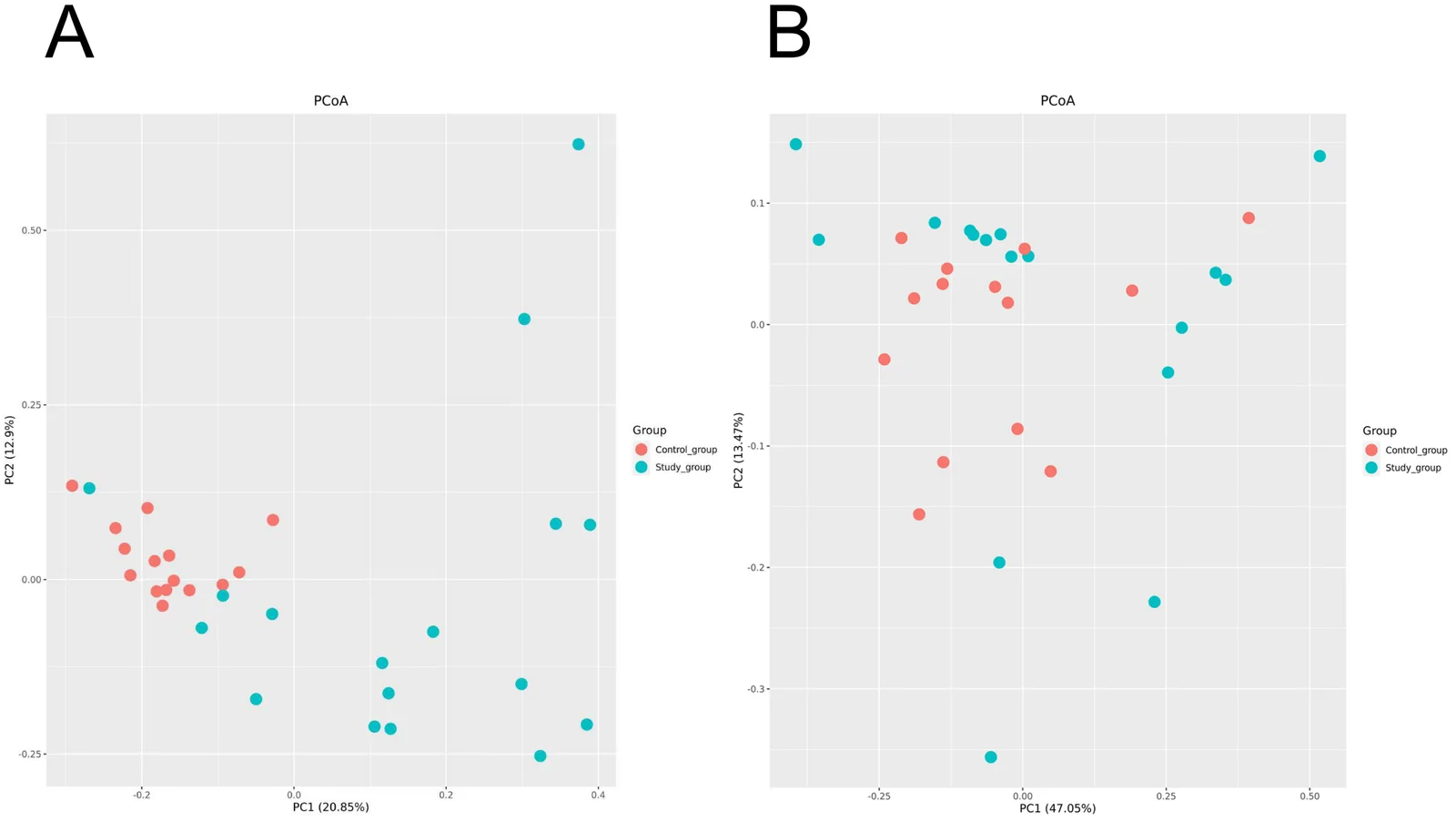
PCoA based on Bray–Curtis dissimilarity at the phylum level showing differences in gut **(A)** and skin **(B)** microbiome composition between patients with acne vulgaris and healthy controls.

Skin microbial beta diversity was assessed using the Bray–Curtis distance metric across multiple taxonomic levels. No statistically significant differences in overall community composition between patients with AV and healthy controls were observed at any taxonomic rank. Bray–Curtis–based comparisons revealed no significant separation between groups at the phylum (*p* = 0.77), class (*p* = 0.975), order (*p* = 0.825), family (*p* = 0.854), genus (*p* = 0.416), or species (*p* = 0.555) levels. Differences in gut (Panel A) and skin (Panel B) microbiome composition between acne patients and healthy controls at the phylum level, assessed by principal coordinates analysis (PCoA) based on Bray–Curtis distances, are shown in **Figure 2**.

### 3.7 Gut microbiome taxonomic composition

**Figure 3:**
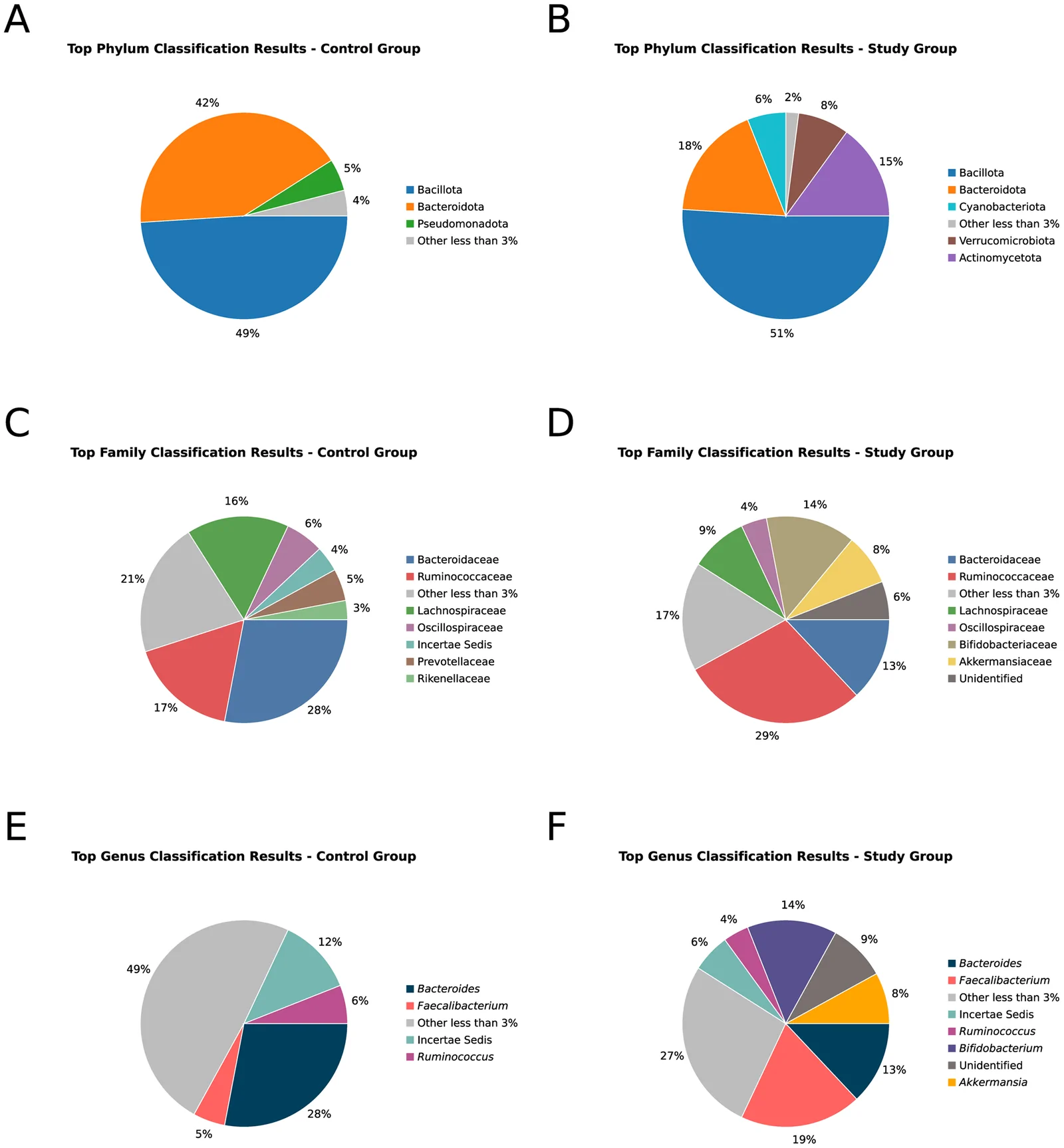
Taxonomic composition of the gut microbiome. Relative abundance of bacterial taxa in fecal samples from patients with acne vulgaris and healthy controls at different taxonomic levels. **(A, B)** present the taxonomic composition at the phylum (L2) level, **(C, D)** at the family (L5) level, and **(E, F)** at the genus (L6) level. Plots illustrate the relative abundance of dominant bacterial taxa in each study group.

At the phylum level, the gut microbiome of both study groups was dominated by Bacillota (formerly Firmicutes). The relative abundance of Bacillota was slightly higher in patients with AV compared with healthy controls (50.6% vs. 48.75%), and this difference was statistically significant (*p* = 0.01). A significantly higher relative abundance of Bacteroidota was observed in healthy controls compared with acne patients (42.55% vs. 18.05%; *p* < 0.001). In the acne group, Actinomycetota (formerly Actinobacteria) accounted for 15.6% of the gut microbiota, whereas this phylum was not detected in the control group; however, this difference did not reach statistical significance (*p* = 0.09). Similarly, Verrucomicrobiota were detected in the acne group (8.02%) but were absent in controls, with the difference approaching but not reaching statistical significance (*p* = 0.06). Additionally, Cyanobacteriota were identified in the acne group (5.89%) but were not detected in the control group; this difference was not statistically significant (*p* = 0.30). At the family level, several statistically significant differences in relative abundance were observed between groups. Bacteroidaceae were significantly more abundant in healthy controls compared with acne patients (28.37% vs. 12.93%; p < 0.001). A significantly higher relative abundance of Lachnospiraceae was also observed in the control group (15.84% vs. 9.21%; p < 0.001), as well as Oscillospiraceae (6.03% vs. 3.76%; p < 0.001). In contrast, Ruminococcaceae were more abundant in patients with AV compared with controls (29.44% vs. 17.31%), with the difference reaching borderline statistical significance (p = 0.054). Rikenellaceae were detected in the control group (3.13%) but were absent in acne patients, and this difference was statistically significant (p = 0.004). Akkermansiaceae were detected exclusively in the acne group (8.01%), whereas they were not observed in controls; however, this difference was not statistically significant (p = 0.09). Similarly, Bifidobacteriaceae were identified in the acne group (14%) but not in the control group, without reaching statistical significance (p = 0.06). No statistically significant differences were observed for Prevotellaceae (p = 0.07). At the genus level, a substantial proportion of sequences remained unclassified, accounting for approximately 49% in the control group and 27.5% in the acne group. Among classified genera, several significant differences were observed between groups. The relative abundance of *Bacteroides* was significantly higher in healthy controls compared with acne patients (28.37% vs. 12.93%; p < 0.001). Similarly, *Ruminococcus* was more abundant in the control group (5.64% vs. 3.82%; p < 0.001). In the acne group, a higher relative abundance of *Akkermansia* was observed (8.01% vs. 0% in controls), and this difference was statistically significant (p = 0.04). A higher proportion of *Faecalibacterium* was also observed in acne patients compared with controls (18.73% vs. 5.18%); however, this difference was not statistically significant (p = 0.25). Likewise, *Bifidobacterium* was detected in the acne group (14%) but not in the control group, without reaching statistical significance (p = 0.09). The relative abundance of gut bacterial taxa at the phylum (L2), family (L5), and genus (L6) levels in acne patients and healthy controls is presented in **Figures 3A–F**. A hierarchical taxonomic tree providing an overview of gut microbial community structure in the control and acne groups is presented in **Supplementary Figures 1A, B**.

### 3.8 Skin microbiome taxonomic composition

**Figure 4:**
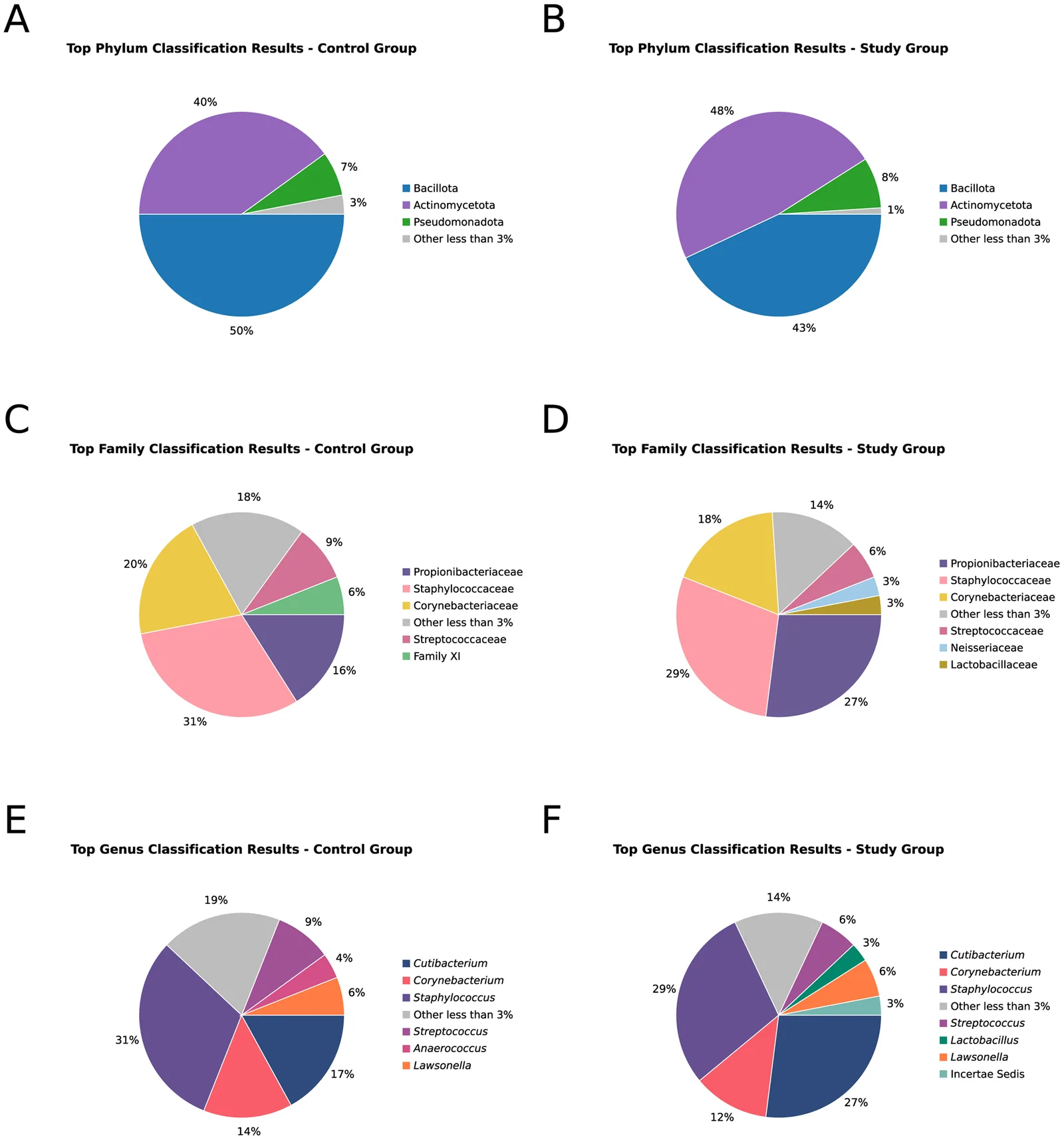
Taxonomic composition of the skin microbiome. Relative abundance of bacterial taxa in skin samples from patients with acne vulgaris and healthy controls at different taxonomic levels. **(A, B)** present the taxonomic composition at the phylum (L2) level, **(C, D)** at the family (L5) level, and **(E, F)** at the genus (L6) level. Plots illustrate the relative abundance of dominant bacterial taxa in each study group.

At the phylum level, the skin microbiome of both patients with AV and healthy controls was dominated by Bacillota. The relative abundance of Bacillota was slightly higher in the control group compared with the acne group (49.93% vs. 43.11%); however, this difference was not statistically significant (p = 0.77). A higher relative abundance of Actinomycetota was observed in patients with acne compared with controls (48.07% vs. 40.27%), although this difference did not reach statistical significance (p = 0.35). Similarly, Pseudomonadota (formerly Proteobacteria) were slightly more abundant in the acne group than in controls (7.5% vs. 6.49%), without statistical significance (p = 0.59). At the family level, Staphylococcaceae predominated in both study groups, accounting for 31.19% of sequences in healthy controls and 29.22% in acne patients, with no statistically significant difference between groups (p = 0.78). The relative abundance of Streptococcaceae was higher in the control group compared with the acne group (9.33% vs. 5.84%), although this difference was not statistically significant (p = 0.66). Conversely, Propionibacteriaceae were more abundant in patients with acne than in controls (26.95% vs. 16.41%), but this difference did not reach statistical significance (p = 0.11). Neisseriaceae (3.4%) and Lactobacillaceae (3.12%) were detected in the acne group but were not observed in the control group; however, these differences were not statistically significant (p = 0.59 and p = 0.36, respectively). Corynebacteriaceae were slightly more abundant in controls compared with acne patients (19.5% vs. 17.56%), without statistical significance (p = 0.63). A substantial proportion of sequences could not be assigned to a specific family, accounting for 17.87% in the control group and 13.91% in the acne group. At the genus level, *Staphylococcus* was the most abundant genus in both groups, representing 31.18% of sequences in healthy controls and 29.21% in patients with acne, with no statistically significant difference (p = 0.79). The relative abundance of *Streptococcus* was higher in controls compared with acne patients (9.26% vs. 5.83%), although this difference was not statistically significant (p = 0.39). *Cutibacterium* was more abundant in the acne group than in the control group (26.95% vs. 16.41%); however, this difference did not reach statistical significance (p = 0.12). *Lactobacillus* was detected in the acne group (3.08%) but not in controls, without statistical significance (p = 0.45). *Corynebacterium* was slightly more abundant in controls compared with acne patients (13.64% vs. 11.48%), with no statistically significant difference (p = 0.87). *Lawsonella* was present at comparable levels in both groups (6.09% in acne patients vs. 5.85% in controls; p = 0.52). A considerable proportion of sequences remained unclassified at the genus level, accounting for 19.36% in the control group and 14.2% in the acne group. The relative abundance of skin bacterial taxa at the phylum (L2), family (L5), and genus (L6) levels in acne patients and healthy controls is illustrated in (**Figures 4A–F**). A hierarchical taxonomic tree illustrating the overall structure of the skin microbiome in both groups is provided in **Supplementary Figures 2A, B**.

## 4 Discussion

Acne vulgaris has traditionally been regarded as a localized disorder of the pilosebaceous unit; however, increasing evidence suggests that systemic factors, including the intestinal microbiome, may play a role in its pathogenesis (Williams et al., 2012; Hazarika, 2021; Lesiak et al., 2024). In this pilot study, we demonstrated pronounced alterations in the gut microbiome of patients with AV, accompanied by more subtle but consistent changes in the skin microbiome, supporting the concept of a functional gut–skin axis and further reinforcing this emerging paradigm. The most robust finding of this study was the significant reduction in gut microbial alpha diversity observed in patients with AV across all applied diversity indices. Reduced gut microbial diversity is widely recognized as a marker of intestinal dysbiosis and has been associated with chronic inflammation, metabolic imbalance, and immune dysregulation (Thursby and Juge, 2017; Gomaa, 2020). Previous studies have reported similar alterations in the gut microbiome of acne patients, including reduced diversity and compositional shifts, supporting the consistency of our findings (Deng et al., 2018; Yan et al., 2018). Beta diversity analysis revealed significant differences in gut microbial community composition between acne patients and healthy controls, indicating structured ecological shifts rather than stochastic variation. Taxonomic analysis revealed an altered balance between major gut bacterial phyla in acne patients, characterized by reduced relative abundance of Bacteroidota and enrichment of Bacillota. Such shifts in gut microbial composition have been associated with low-grade systemic inflammation, altered lipid metabolism, and insulin resistance, processes that may contribute to acne pathophysiology (Ley et al., 2006; Delzenne et al., 2011). Moreover, the decreased abundance of bacterial families such as Bacteroidaceae and Lachnospiraceae, which are involved in short-chain fatty acid production and maintenance of intestinal barrier integrity, may further promote systemic inflammatory responses relevant to AV development (Parada Venegas et al., 2019; Vacca et al., 2020). As described in Section 3.7 (Gut microbiome taxonomic composition), members of the family Bifidobacteriaceae were detected exclusively in the acne group (14%), although this difference did not reach statistical significance. This observation should be interpreted with caution. Both *Bifidobacterium* and *Cutibacterium* belong to the phylum Actinomycetota and diverge at lower taxonomic ranks. It is well established that 16S rRNA gene sequencing, particularly when targeting the V3–V4 hypervariable regions, may have limited discriminatory power for closely related taxa (Yarza et al., 2014; Johnson et al., 2019). Taxonomic assignment is further influenced by the choice of reference database and classifier training strategy, including SILVA-based annotations (Edgar, 2018). Therefore, although the presence of Bifidobacteriaceae in acne patients may reflect genuine alterations in gut microbial composition, potential misclassification among phylogenetically related Actinomycetota taxa cannot be entirely excluded. Confirmation using higher-resolution approaches such as shotgun metagenomics would help clarify this finding (Quast et al., 2013). In contrast to the pronounced alterations observed in the gut microbiome, changes in the skin microbiome were more modest. Although alpha diversity indices reflecting both richness and evenness were significantly reduced in acne patients, beta diversity analysis did not reveal significant differences in overall skin microbial community structure. This pattern suggests that acne-related skin dysbiosis is characterized primarily by shifts in relative abundance and dominance of specific taxa rather than by the emergence of entirely distinct microbial communities (Grice and Segre, 2011; Kong and Segre, 2012; Fitz-Gibbon et al., 2013; O'Neill and Gallo, 2018). Previous studies have shown that acne is not driven solely by the presence of *C. acnes*, but rather by imbalances in its relative abundance, strain diversity, and interactions with other commensal skin microorganisms (Fitz-Gibbon et al., 2013; Sanford and Gallo, 2013; Dréno et al., 2018; O'Neill and Gallo, 2018). The absence of significant beta diversity differences in the present study is therefore consistent with the concept of acne as a state of ecological imbalance within an otherwise conserved skin microbial ecosystem (Kong and Segre, 2012). In several analyses, marked differences in the relative abundance of specific taxa were observed between acne patients and healthy controls without reaching statistical significance. This likely reflects high inter-individual variability combined with the limited sample size inherent to the pilot design of this study. In microbiome research, certain taxa may be absent in a proportion of individuals while occurring at high abundance in a subset, resulting in wide dispersion of relative abundance values and reduced statistical power despite large mean differences (Lozupone et al., 2012). Moreover, the compositional nature of microbiome data constrains statistical inference, as relative abundance values are interdependent and influenced by shifts in other taxa within the microbial community (Gloor et al., 2017). The application of centered log-ratio transformation and ALDEx2 provides a conservative analytical framework that minimizes false-positive findings but may reduce sensitivity for detecting differences in low-prevalence or highly variable taxa, particularly in small cohorts (Fernandes et al., 2014; Weiss et al., 2017). The parallel reduction in alpha diversity observed in both gut and skin microbiomes supports the hypothesis that systemic dysbiosis may influence local skin microbial homeostasis. The gut–skin axis describes bidirectional interactions between the intestinal microbiome, immune system, metabolic pathways, and skin physiology (Salem et al., 2018; De Pessemier et al., 2021; Mahmud et al., 2022). Alterations in gut microbial diversity and composition may affect cutaneous homeostasis indirectly through increased intestinal permeability, immune modulation, and systemic low-grade inflammation, thereby influencing inflammatory responses within the skin (Belkaid and Hand, 2014; Bischoff et al., 2014). In this context, gut dysbiosis may act as an upstream factor that predisposes to cutaneous dysbiosis by shaping host immune responses, metabolic signaling, and barrier function, rather than directly altering skin microbial composition. This concept is consistent with the present findings, in which pronounced alterations in gut microbial diversity were accompanied by more modest but concordant changes in the skin microbiome. Such a hierarchical relationship between gut and skin microbial ecosystems has been proposed in AV and other inflammatory skin disorders, supporting a systemic contribution to disease pathogenesis (Deng et al., 2018; Salem et al., 2018). These findings should be considered in light of the exploratory nature of this study. While the sample size may have limited the detection of subtle differences, particularly among low-abundance skin-associated taxa, the applied approach enabled a comprehensive characterization of both gut and skin microbiomes. Despite predefined exclusion criteria, residual confounding related to diet, body mass index, or short-term medication exposure cannot be fully excluded. Moreover, although 16S rRNA gene sequencing does not provide strain-level or functional resolution, it represents a robust and widely accepted method for profiling microbial community structure. Future studies involving larger, longitudinally followed patient cohorts and advanced molecular approaches, such as shotgun metagenomics, metabolomic profiling, and integration with detailed clinical phenotyping, may provide mechanistic insight into how microbiome alterations influence AV development, disease course, and treatment response. Such integrative strategies have the potential to identify microbiome-based biomarkers and therapeutic targets, paving the way toward more personalized approaches to AV management. One of the principal limitations of the present study is that only the bacterial component of the skin and gut microbiome was evaluated using 16S rRNA gene sequencing. We acknowledge that the human microbiome also comprises fungi, viruses, archaea, and other microorganisms, all of which may contribute to host–microbiome interactions. Although most published studies on the microbiome in AV have focused primarily on bacterial communities using 16S rRNA sequencing, increasing evidence suggests that integrated multi-kingdom microbiome analyses may provide a more comprehensive understanding of acne pathogenesis. Characterization of the mycobiome and virome requires dedicated sequencing strategies, including ITS sequencing for fungi and metagenomic approaches for viruses, which were not included in the present study design. Therefore, future investigations should integrate bacterial, fungal, and viral profiling to better characterize the complex microbial ecosystem associated with AV.

## Data Availability

The data presented in the study are deposited in the Jan Kochanowski University repository, at this link: https://repozytorium.ujk.edu.pl/publication/14628.
