# Supplementary material for: Gut and skin dysbiosis in acne vulgaris: a comparative microbiome analysis
Source: Front Cell Infect Microbiol. 2026 Aug 6;16:1904271. doi: 10.3389/fcimb.2026.1904271 (PMC13489829; doi:10.3389/fcimb.2026.1904271)
Supplement: Supplementary file 2 [file DataSheet2.pdf]

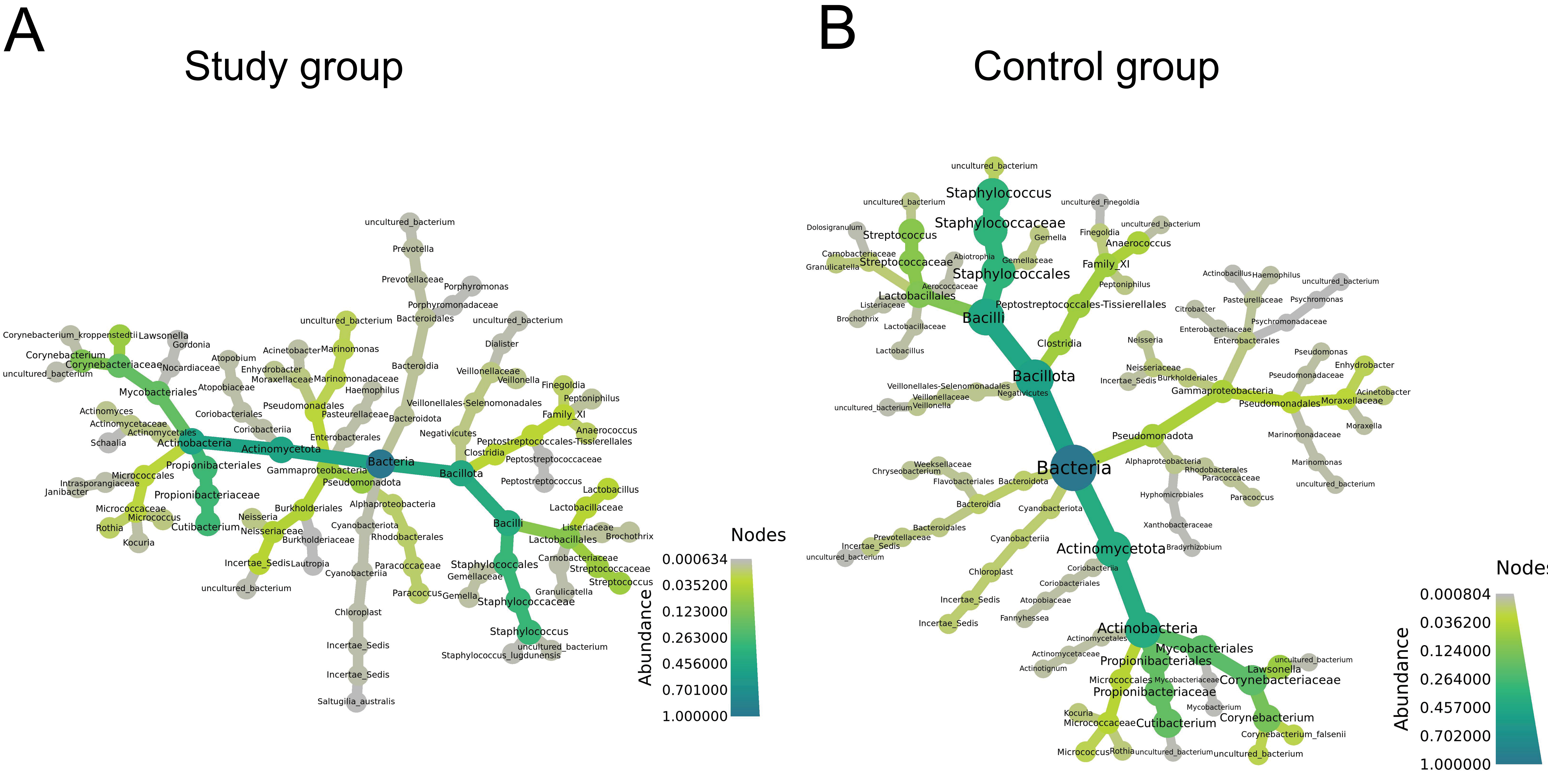

Supplementary Figure 2. Heat-tree visualization of the skin microbiome showing taxonomic structure and relative abundance of bacterial taxa in control subjects and acne patients. Branch thickness and color intensity correspond to relative taxon abundance.
